# Rumen bacteria, feed utilization, and milk production of Damascus goats fed different levels of azolla meal

**DOI:** 10.1038/s41598-026-38113-6

**Published:** 2026-04-23

**Authors:** Ahmed F. A. Abd-Elgwad, Salah Abo Bakr, Ebrahim A. Sabra, Mahmoud M. Khorshed, Hamdy M. Metwally, Alaa Emara Rabee

**Affiliations:** 1 Animal and Poultry Nutrition Department, Desert Research Center, Cairo, Egypt; 2 Genetic Engineering and Biotechnology Research Institute, University of Sadat City, Sadat, Egypt; 3 Animal Production Department, Faculty of Agriculture, Ain-Shams University, Cairo, Egypt

**Keywords:** Azolla, Lactating goats, Rumen microbiome, Rumen fermentation, Nutrient digestibility, Milk yield, Feed efficiency, Biotechnology, Microbiology, Zoology

## Abstract

Azolla could be a promising alternative feed as it is a rich source of nutrients and bioactive compounds that can modulate rumen microbiota and improve animal productivity. This study evaluated the effects of inclusion dietary Azolla (*Azolla pinnata*) as a partial replacement of concentrate feed mixture (CFM) on rumen bacteria, nutrient digestibility, and milk production in lactating Damascus goats. Thirty-two goats were assigned to four groups (n = 8): a control group (C), A10, A20, and A30 to receive 0, 10%, 20%, and 30% of Azolla as a replacement of CFM, respectively. Microbial diversity increased in Azolla-fed goats, with enrichment of phylum Firmicutes in Azolla-supplemented groups C=13.36%, A10= 31.09%, A20= 25.15%, and A30= 29.85%. Fiber-degrading bacterial genera such as *Prevotella*, *Ruminococcus*, and *Christensenellaceae R-7 group*. .Crude protein digestibility was declined in supplemented groups and was found in order 74.76>69.11>68.24>63.93 %. Total volatile fatty acids (TVFA) and the concentration of acetate, propionate, and butyrate were higher in supplemented groups (p < 0.01). Fat-corrected milk (FCM) was higher in A20 (1139 mL/head) compared to other groups (C=992, A10=1050, A30=888 mL/head and feed efficiency followed the same trend (p < 0.05). Azolla could replace 20% of CFM in goats’ diet, and it is a viable alternative feed resource, particularly under challenges associated with the availability of conventional concentrate mixtures.

**Supplementary Information:**

The online version contains supplementary material available at 10.1038/s41598-026-38113-6.

## Introduction

Goats are well adapted to arid conditionsandhave higher efficiently utilize low-quality forages and maintain productivity under harsh conditions, making them a valuable livestock species for food security in developing regions^1–3^.

The productivity of ruminants rely on microbial rumen fermentation, that is driven by bacteria, protozoa, fungi, and archaea. This microorganisms ferment fibrous plant materials into microbial proteinand volatile fatty acid (VFA) which serve as the primary sources of protein and energy for the host animal^1,2^. Consequently, animal performance is linked to ruminal microbial fermentations, which are influenced by host species and diet composition. Understanding the changes in rumen microbiota due to dietary interventions provides opportunities to enhance feed utilization, animal productivity, and mitigate methane emissions^4^.

The prohibition of antimicrobial growth promoters has directed attention to plant-derived phytochemicals such as polyphenols, saponins, and essential oils, which modulate rumen microbiome and improve feed utilization andanimal health^5,6^. Additionally, livestock sector faces increasing challenges in the availability of feed resources due to climate change, andrising feed costs^7^. These circumstances highlight the urgent need to explore non-traditional feed resources such as Azolla to sustain productivity while reducing environmental impacts. Thus, combining the adaptive capacity of goats with alternative feed resources, productivity can be sustained while reducing environmental impacts and nutrition cost.

Azolla, a small aquatic fern hosting the nitrogen-fixing cyanobacterium, *Anabaena azollae*. Azolla is rich in protein (13–30%), essential amino acids, vitamins (β-carotene, A, B12), minerals, and antioxidants, making it a highly nutritious feed alternative^8–10^. Dietary inclusion of dried Azolla at 20% of diet enhanced feed efficiency, milk yield, and milk fat in lactating Zaraibi goats Hassanein et al.,^11^. Similar conclusion was obtained on Barbari goats Chaudhary et al,^12^**.**

Beyond its nutritional value, Azolla contains bioactive phytochemicals, including polyphenols, flavonoids, and saponins, which modulate rumen microbial activities and improve fermentation efficiency^9,10^. Nevertheless, evidence regarding the direct impact of Azolla on the rumen microbiome and fermentation dynamics remains limited. Azolla supplementation (10–20%) improved the in vitro digestibility of nutrients and VFA production, and increased the abundance of fibrolytic bacteria such as *Prevotella*, *Rikenellaceae RC9 gut group*, and *Christensenellaceae R-7 group*, enhancing volatile fatty acid production and nutrient digestibility in ruminants^10^**.** However, in vivo evidence the effects of Azolla on rumen microbiota in goats remains limited. Plant-derived phytochemicals, such as polyphenols, saponins, and essential oils, further support microbial modulation and animal health^5,6^. Dietary phytochemicals enhanced the ruminal fiber-degrading bacteria and improved volatile fatty acids (VFA) production in goats^13^**.**

Further research is needed to clarify the effect of azolla supplementation level on the changes in rumen microbiota and their association with animal performance. This study addresses the current knowledge gap by evaluating the effect of dietary Azolla supplementation level on rumen bacteria, fermentation characteristics, nutrient digestibility, and milk yield in Damascus goats.

## Materials and methods

### Ethics

The use of animals of goats in this study was reviewed and approved by the Institutional Animal Care and Use Committee of the Desert Research Center (DRC), Egypt (Approval No.: 51053).

### Animals, diets, and experimental design

All experimental protocols and methods, and the use of animals were approved by and followed the guidelines and regulations of the Institutional Animal Care and Use Committee of the Desert Research Center (DRC) (Approval No.: 51053), Egypt, and followed the guidelines and regulations of ARRIVE 2.0 protocol and the European Union standards for animal research. This study was carried out at Maryout Research Station, DRC, Egypt (30 km from Alexandria, Egypt). Thirty-two lactating goats (6-8 years old, 38.5±1.5 Kg average body weight, and similar parity number (5-6 kidding) in the initial lactation period (20 ± 7 days after kidding) were used in this 100-day study and were randomly divided into four similar groups (n=8)The sample size was determined based on the availability of goats exhibiting comparable physical and physiological characteristics. All the goats were healthy and free of diseases. The goats used in this experiment are the offspring of the goat herd in Maryout Research Station, DRC, Egypt and were included in the study after obtaining approval from the administration of Maryout Research Station and the Animal and Poultry Production Division, DRC. Dry Azolla was obtained from Azolla Modern Feed Company (Belbeis–10^th^ of Ramadan desert road, Sharqia, Egypt). Azolla cultivation, management, and harvesting followed the method described in Abd-Elgwad et al^9^. All goats were fed the same basal diet consisting of 60% concentrate feed mixture (CFM) and 40% Egyptian clover hay (*Trifolium alexandrinum*) to meet their nutritional requirements according to Keral^11^. The four experimental diets differed in the levels of dried azolla included in the CFM (Table 1): a group received the control diet with 10% Azolla from the CFM (A10); a group received the control diet with 20% Azolla from the CFM (A20), and a group received the control diet with 30% Azolla from the CFM (A30).The inclusion levels of Azolla were determined according to the recommendations of an in vitro study by Abd-Elgwad et al^10^.

**Table 1:** The ingredients of concentrate feed mixtures and chemical composition of experimental diets.

	Concentrate				Azolla	Berseem hay
	**C**	**A10**	**A20**	**A30**		
Yellow corn	52.00	52.00	52.00	52.00	-	-
Soybean meal	12.00	12.00	12.00	12.00	-	-
Wheat brane	20.00	14.00	7.00	0.00	-	-
Cotton seed meal	10.00	6.00	3.00	0.00	-	-
Azolla	0.00	10.00	20.00	30.00	-	-
Molasses	2.00	2.00	2.00	2.00	-	-
Salt	1.20	1.20	1.20	1.20	-	-
Calcium carbonate	2.00	2.00	2.00	2.00	-	-
Yeast	0.20	0.20	0.20	0.20	-	-
Vitamins and minerals	0.20	0.20	0.20	0.20	-	-
Antitoxins	0.20	0.20	0.20	0.20	-	-
Bicarbonate	0.20	0.20	0.20	0.20	-	-
Chemical composition, %						
DM	93.00	92.00	91.00	90.00	92.00	92.00
OM	93.00	90.00	88.00	86.00	75.80	88.00
CP	17.00	16.40	16.20	16.00	17.12	15.08
EE	4.68	3.96	4.13	4.54	3.84	3.23
CF	6.64	7.06	8.26	9.56	16.53	31.40
Ash	7.00	10.00	12.00	14.00	24.20	12.00
NDF	35.00	40.00	45.00	51.00	48.37	54.93
ADF	16.10	18.00	20.00	21.00	31.08	41.10
NFE	64.68	62.58	59.41	55.9	38.31	38.29
GE Mcal/kg DM	446.22	428.73	420.90	414.34	370.68	413.83

DM= Dry matter; OM= organic matter; CP=crude protein; EE= ether extract; CF=Crude fiber;

NDF= neutral detergent fiber; ADF= Acid detergent fiber; C=control diet;

A10= Diet supplemented with 10% azolla; A20= Diet supplemented with 20% azolla;

A30: Diet supplemented with 30% azolla.

NFE (%)=100−(CP+CF+EE+Ash),

GE: Gross energy (Mcal/kg DM): CP×5.65+ CF×4.15+EE×9.40+NFE×4.15^23^.

The goats were housed in shaded pens (40 m^2^ per group) and had free access to clean drinking water. Body weights were recorded at both the beginning and every 15 days. Feed was offered twice daily at 08:00 and 12:00 h, and feed refusals were weighed daily to monitor intake. Table 1 presents the ingredients and chemical composition of the diets, including DM, OM, CP, EE, CF, Ash, NDF, ADF, NFE, and GE. The experimental diets conatined approximately similar in CP content (CP ≈ 16–17%). Feed refusals were weighed, and daily feed intake was recorded. Representative samples of both the offered and refused diets were collected weekly and dried in a forced-air oven at 65 °C for 48 h. This experiment did not involve any clinical trials or animal euthanasia. Upon completion of the study, all goats were returned to the goat herd.

### Rumen sampling and fermentation parameters

By the end of the experiment, rumen contents were withdrawn from the goats , four hours after morning feeding. Rumen samples were collected from the goats using stomach tubing with a standardized tube depth across all goats. The initial portion of rumen fluid was discarded to minimize saliva contamination.. The collected samples were filtered through four layers of cheesecloth to remove feed particles and obtain a representative rumen fluid sample and rumen pH was measured immediately after sample collection (WPA CD70, ADWA, Szeged, Hungary).The filtered rumen samples were used to determine volatile fatty acids (VFAs) and ammonia nitrogen (NH₃N) concentrations, as well as for microbial DNA isolation. The quantification of rumen VFAs followed the procedures described by Rabee et al^13^. Briefly, 1 mL of rumen fluid was acidified with 200 μL of 25% metaphosphoric acid, then centrifuged at 13,000 rpm for 14 min. The resulting supernatant was used for VFA and ammonia determination.

Ammonia concentration was measured using the standard Kjeldahl steam distillation method using a VELP UDK 139 semi-automatic system (VELP Scientifica, Italy)^14^. VFA concentrations were analyzed using a TRACE 1300 gas chromatography system (Thermo Fisher Scientific, Waltham, MA, USA) equipped with a TR-FFAP capillary column (30 m × 0.53 mm i.d., 0.5 μm film thickness)^13^. Nitrogen was used as the carrier gas at a flow rate of 7 mL/min, while hydrogen and make-up gases were supplied at 40 and 35 mL/min, respectively. The total run time was 10 min, and calibration was performed using standard solutions containing known VFA concentrations. In addition, the predicted methane concentration was estimated based on propionic acid concentration using the following equation: Methane yield = 316 / propionate + 4.4^15^.

### Digestibility trial

Digestibility trial was conducted at the end of the lactation trial . Goats were placed in metabolism cages for 14 days, including seven days for adaptation followed by seven days for fecal collection, to evaluate the effects of graded levels of Azolla inclusion (0–30%) on feed intake and nutrient digestibility. All goats were weighed at the beginning and end of the trial, and the daily feed offered and refused was recorded throughout the experimental period.

During the collection period, the total daily fecal output of each goat was collected, mixed thoroughly, and measured daily. Then, a 10% subsample of the total feces from each animal was collected. The quantities of feed offered, refused, and water intake were also measured and recorded daily. Subsamples of feces, feeds, and refused feeds were pooled for each animal over the seven-day collection period^16^. These samples were dried at 65 °C for 48 h to a constant weight, ground, and stored for subsequent chemical analysis. Nutrient digestibility coefficients were determined according to the procedures described by McDonald et al^16^. Digestible crude protein (DCP) and total digestible nutrients (TDN) were calculated using standard equations of **NRC**^17^:

$$\text{DCP (\% of DM)}=\text{CP (\% of DM)}\times \text{digestibility coefficient}$$

$$\text{TDN (\% of DM)}={\mathrm{DCP}}+\text{digested fiber fraction}+(\text{EE digested fraction}\times 2.25)$$

### Milk production and composition, and feed efficiency

Milk yield was individually recorded on days 20, 35, 45, and 60 of lactation by hand milking twice daily (morning and evening) by the same technician to minimize human error. Representative milk samples were collected from morning and evening milking in proportion to their respective milk yields and the samples were pooled to combine one representative sample for the subsequent analyses. The kids were separated from their dams for 12 h prior to milking. Milk composition (total solids, total nitrogen, ash, and solid-not-fat) was analyzed according to AOAC^16^, where total nitrogen was determined using the micro-Kjeldahl method, ash content by muffle furnace (Thermolyne type 1500), fat content by the modified method of Singh and Pratap^19^, and total carbohydrates were calculated by difference.

Gross feed efficiency (FE) was calculated as the ratio between daily milk yield (g/day) and dry matter intake (DMI, kg/day). To standardize milk yield based on fat content, the 3.5% fat-corrected milk (FCM) was computed following the equation proposed by Sklan et al^20^: 3.5% FCM (kg/day) = (0.432 + 0.1625 × % milk fat) × actual milk yield (kg/day). Thereafter, the adjusted feed efficiency was expressed as the ratio of 3.5% fat-corrected milk yield (g) to the corresponding dry matter intake (kg), providing a more accurate estimate of feed utilization efficiency among treatments.

### Microbial community

#### DNA extraction and PCR amplification

Rumen microbial DNA was extracted from 750 µL of rumen fluid followed by centrifugation at 13,000 rpm for 15 minutes to obtain microbial pellets. The extraction was performed using the QIAamp DNA Stool Mini Kit (Qiagen, Hilden, Germany), and the purified DNA was eluted in 50 µL of elution buffer. The concentration and integrity of the extracted DNA were evaluated through a NanoDrop 2000 spectrophotometer (Thermo Scientific, Massachusetts, USA) and agarose gel electrophoresis. The bacterial community structure was characterized by amplifying the V4 hypervariable region of the 16S rDNA gene with the primer pair 515F/926R. PCR amplification was carried out under the following conditions: an initial denaturation at 94 °C for 3 min, followed by 35 cycles of denaturation at 94 °C for 45 s, annealing at 50 °C for 60 s, and extension at 72 °C for 90 s, ending with a final extension step at 72 °C for 10 min. The resulted PCR amplicons were purified, quantified, and subjected to paired-end sequencing on the Illumina MiSeq platform at the Integrated Microbiome Resource (IMR), Dalhousie University, Halifax, Canada, to profile the rumen bacterial communities.

#### Bioinformatics analysis

The bioinformatics workflow followed the approach described by Rabee et al^13^. In brief, paired-end raw reads (Average 91564 ±9976 sequence reads per sample) were processed using the DADA2 pipeline in R (v3.5.2)^21^. Raw fastq files were demultiplexed, quality-checked, filtered, trimmed, and dereplicated. Forward and reverse reads were then merged to generate denoised sequences, retaining high-quality samples with Phred scores ≥30 for the subsequent analysis. Chimeric sequences were removed to obtain amplicon sequence variants (ASVs)^13,21^. Taxonomic classification of ASVs was performed using the assignTaxonomy and assignSpecies functions against the SILVA v138 reference database. Alpha diversity indices (Observed ASVs, Chao1, Shannon, and Inverse Simpson) were computed to assess microbial richness and evenness, while beta diversity was evaluated using Bray–Curtis dissimilarity and visualized by PCoA plots generated via the phyloseq and ggplot2 packages [13,121]. Raw sequence data have been deposited in the NCBI SRA under accession number: PRJNA1291228.

#### Proximate chemical analysis and phytochemical compounds in animal feeds

Dried Azolla, feed, and fecal samples were finely ground and analyzed according to AOAC^18^ methods to determine dry matter (DM; 930.15), crude protein (CP; 954.01),crude fiber (CF: method 978.10), and ether extract (EE; 920.39). Neutral detergent fiber (NDF) and acid detergent fiber (ADF) were measured using an ANKOM200 Fiber Analyzer with commercial kits (ANKOM Technology, NY, USA) following the procedure of Van Soest et al.^22^. Gross energy (GE) was calculated from chemical composition according to Blaxter^23^, and nitrogen-free extract (NFE) was estimated by difference, as described in Table 1.

Total phenols, flavonoids, and tannins in the feed samples were quantified using colorimetric methods described in previous studies^24–26^. Individual phenolic compounds were identified by HPLC (Thermo Scientific, USA) equipped with a C18 column and a mobile phase of water and 0.05% trifluoroacetic acid in acetonitrile at a flow rate of 0.9 mL/min^27^.

### Statistical analysis

Data on the relative abundances of microbial groups were tested for normality and homogeneity by the Shapiro–Wilk test, and non-normal variables were then arcsine transformed. The effects of Azolla inclusion levels (independent variable) on the differences in feed intake, nutrient digestibility, rumen fermentation traits, bacterial diversity and relative abundance, and milk yield (dependent variables) were analyzed using one-way ANOVA (${Y}_{ij}=\mu +{T}_{i}+{e}_{ij}$), with linear and quadratic trend analyses to assess response patterns. Significant differences among means were further evaluated using Duncan’s multiple range test at p < 0.05. To explore the influence of Azolla supplementation on the overall response patterns, principal component analysis (PCA), permutational multivariate analysis of variance (PERMANOVA), and correlation analysis were applied to using data of milk yield, digestibility, rumen fermentation, and microbial composition. All statistical analyses were carried out using SPSS software (version 20.0; IBM, USA)^28^ and PAST software^29^.

## Results

### Chemical composition and phytochemical content of experimental diets

Azolla had comparable CP to CFMs and was higher than Berseem hay, while CFMs were richer in EE, and BH contained more fiber (CF, NDF, ADF). Ash content was higher in Azolla (Table 1). The phytochemical substances of Azolla, CFM, and hay are presented in Supplementary Table S1. Azolla exhibited significantly higher concentrations of total polyphenols (5%) and total tannins (4.31%) compared to CFM (polyphenols= 0.05%, tannins= 0.17%) and BH (polyphenols= 0.09 %, tannins= 0.18%). On the other hand, Total flavonoids was higher in CFM ( 0.40% ) and BH (0.31%) than Azolla (0.23%). As a result of its high total polyphenols and flavonoids contents, Azolla exhibited significantly higher levels of most phenolic compounds compared to BH and CFM.

### Microbial diversity

**Fig. 1:**
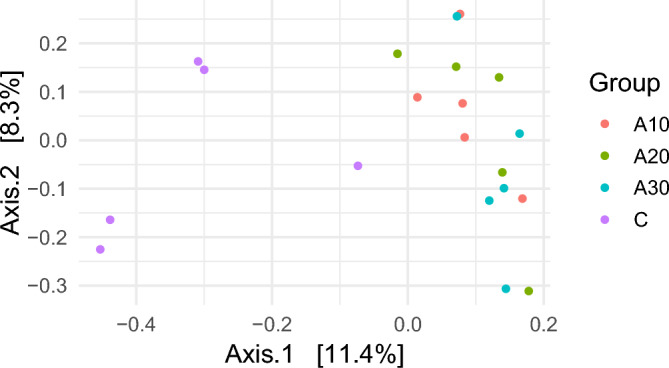
Principal coordinates analysis (PCoA) of the bacterial community using Bray-Curtis dissimilarity. The analysis was conducted between four lactating goat groups: Purple circles for goats supplemented with a control diet (C), red circles for goats supplemented with 10% azolla (A10), green circles for goats supplemented with 20% azolla (A20), and blue circles for goats supplemented with 30% azolla (A30).

The microbial alpha diversity indices ASVs, Chao1, Shannon, and Inverse Simpson are summarized in Table 2. Shannon index showed a significant increase (p < 0.05) in all due to Azolla supplementation and was found in order 2.84<4.98<4.70<5.11for groups C, A10, A20, and A30, respectively. (Table 2; Supplementary Figure S1). Furthermore, beta diversity analysis, visualized by principal coordinate analysis (PCoA) based on Bray-Curtis dissimilarity, demonstrated a clear separation between the control (C) and Azolla-treated groups (A10, A20, and A30) (Figure 1). This distinct clustering pattern suggests that Azolla inclusion induced noticeable alterations in the overall rumen bacterial community structure.

**Table 2:** Effect of Azolla supplementation on bacterial diversity and the relative abundances (%) of bacterial phyla in the rumen of lactating goats.

	**C**	**A10**	**A20**	**A30**	**SEM**	**p- value**
	**Mean**	**Mean**	**Mean**	**Mean**		
Observed ASVs	259.80	375.40	323.60	417.60	23.05	0.07
Chao1	285.27	389.22	332.59	438.52	24.89	0.13
Shannon	2.84^a^	4.98^b^	4.70^b^	5.11^b^	0.22	0.0001
Inverse Simpson	48.82	74.6	53.97	85.23	5.83	0.07
Bacterial phyla, %						
Actinobacteriota	0.32	0.00	0.02	0.00	0.00	0.00
Armatimonadota	0.00	0.01	0.00	0.01	0.00	0.00
Cyanobacteria	0.85^b^	0.08^a^	0.18^a^	0.44^a^	0.09	0.004
Bacteroidota	64.34	67.2	71.04	68.53	0.93	0.06
Desulfobacterota	0.17^c^	0.07^ab^	0.12^bc^	0.026^a^	0.02	0.012
Elusimicrobiota	0.02	0.03	0.03	0.02	0.00	0.104
Fibrobacterota	0.00	0.03	0.00	0.02	0.00	0.00
Firmicutes	13.36^a^	31.09^c^	25.15^b^	29.85^c^	1.73	0.0001
Planctomycetota	0.64^b^	0.08^a^	0.04^a^	0.05^a^	0.06	0.0001
Proteobacteria	13.89^b^	0.03^a^	0.08^a^	0.08^a^	1.43	0.0001
Spirochaetota	5.51^b^	0.86^a^	2.00^a^	0.51^a^	0.62	0.006
Synergistota	0.31	0.06	0.46	0.00	0	0.00
Verrucomicrobiota	0.20^ab^	0.15^a^	0.33^b^	0.12^a^	0.03	0.027

C= goats supplemented with control diet; A10= goats supplemented 10% azolla; A20= goats supplemented with 20% azolla; A30= goats supplemented with 30% azolla; ASVs= Amplicon Sequence Variants; ^a,b,c,d^ Means within a row with different subscripts differ significantly (p < 0.05). SEM= Standard error of means.

### Structure of bacterial community

Taxonomic analysis of the rumen bacterial community revealed 13 phyla. Bacterial community was dominated by Bacteroidota (67.78%), Firmicutes (24.8%), Spirochaetota (2.23%), and Proteobacteria (3.52%). Bacterial phyla that represent less than 1% of the bacterial community were Cyanobacteria, Desulfobacterota, Elusimicrobiota, Planctomycetota, and Verrucomicrobiota(Table 2).

Phylum Bacteroidota predominated the bacterial community (Table 2). This phylum is classified into families Rikenellaceae and Prevotellaceae, F082, Muribaculaceae, Bacteroidales RF16 group, Bacteroidales BS11 gut group, and p-2534-18B5 gut group. The Rikenellaceae family comprised three genera *U29-B03*, *SP3-e08*, and *Rikenellaceae RC9 gut group* (Table 3). Genus *Rikenellaceae RC9 gut group* had significantly higher relative abundance (34.65%) in the control group (C) (p < 0.05), followed by A20 (24.59%), A10 (19.5%), and the lowest in A30 (18.46%). Meanwhile, among the family Prevotellaceae, genus *Prevotella* was the most dominant, showing a significant increase (p < 0.05) in Azolla-supplemented groups (A10= 26.18%, A20=29.09 %, A30= 30.44% ) compared to the control (15.90 %) (Table 3). Family F082 showed a significantly higher abundance (p < 0.05) in the A10 group (14.84%) than in other treatments (A20= 8.39%, A30 =9.5%) and the control (10.12%) (Table 3). Families p-2534-18B5 gut group and Muribaculaceae were higher in A10 and A30 compared to other groups (p<0.05). Family Bacteroidales RF16 group showed its higher relative abundance in A20 (2.57%), followed by A30 (1.13%), A10 (0.77%), C (0.73%) respectively (p<0.05) (Table 3). Moreover, family Bacteroidales BS11 gut group had proportion in group A20 (3.08%) compared to C (1.50%), A10 (1.50%), and A30 (0.97%)(p<0.05) (Table 3).

**Table 3:** Effect of Azolla supplementation on the relative abundances (%) of dominant bacterial families and genera in the rumen of lactating goats.

	**CC**	**A10**	**A20**	**A30**	**SEM**	**p- value**
	Mean	**Mean**	**Mean**	**Mean**		
P: Bacteroidota; F: Rikenellaceae						
*F: Rikenellaceae*	34.96^c^	19.78^a^	24.77^b^	19.14^a^	1.67	0.0001
*G: U29-B03*	0.03	0.03	0.01	0.02	0.00	0.27
*G: SP3-e08*	0.21	0.44	0.12	0.58	0.07	0.10
*G: Rikenellaceae RC9 gut group*	34.65^c^	19.25^ab^	24.59^b^	18.46^a^	1.70	0.0001
P: Bacteroidota; F: Prevotellaceae						
F: Prevotellaceae	16.37^a^	27.47^b^	31.36^b^	33.38^b^	2.05	0.005
*G: Prevotella*	15.90^a^	26.18^b^	29.09^b^	30.44^b^	1.84	0.009
*G: Prevotellaceae UCG-003*	0.20^a^	0.55^a^	1.60^b^	2.05^b^	0.21	0.0001
*G: Prevotellaceae UCG-001*	0.20	0.29	0.43	0.24	0.05	0.48
F: F082	10.12^a^	14.84^b^	8.39^a^	9.50^a^	0.66	0.02
F: Muribaculaceae	0.13^a^	0.29^b^	0.10^a^	0.32^b^	0.03	0.014
F: Bacteroidales RF16 group	0.73^a^	0.77^a^	2.57^c^	1.13^b^	0.24	0.006
F: Bacteroidales BS11 gut group	1.50^a^	1.50^a^	3.08^b^	0.97^a^	0.31	0.008
F: p-2534-18B5 gut group	0.05^a^	1.66^b^	0.11^a^	1.75^b^	0.40	0.02
P: Firmicutes; F: Christensenellaceae						
F: Christensenellaceae	2.14	3.44	3.47	3.04	0.23	0.14
*G: Christensenellaceae R-7 group*	1.88^a^	2.95^b^	2.97^b^	2.60^b^	0.22	0.045
P: Firmicutes; F: Ruminococcaceae						
F: Ruminococcaceae	1.88^a^	13.80^b^	13.17^b^	12.61^b^	1.25	0.0001
*G: Ruminococcus*	0.95^a^	2.12^b^	2.18^b^	1.76^b^	0.32	0.045
*G: unclassified__Ruminococcaceae*	0.93^a^	11.68^b^	10.99^b^	10.86^b^	1.12	0.0001
P: Firmicutes; F: Oscillospiraceae						
F: Oscillospiraceae	2.16^a^	3.41^ab^	2.30^a^	4.42^b^	0.30	0.01
*G: NK4A214 group*	0.84^a^	1.83^ab^	1.20^a^	2.86^b^	0.26	0.01
P: Firmicutes; F: Hungateiclostridiaceae						
F: Hungateiclostridiaceae	0.44^b^	0.49^b^	0.21^a^	0.26^a^	0.04	0.003
*G: Saccharofermentans*	0.35^b^	0.32^b^	0.14^a^	0.16^a^	0.03	0.005
*P: Firmicutes; F: Lachnospiraceae*						
*F: Lachnospiraceae*	1.86^a^	2.94^bc^	2.32^ab^	3.29^c^	0.18	0.008
*G: Butyrivibrio*	0.32	0.28	0.38	0.50	0.05	0.55
*G: Lachnospiraceae NK3A20 group*	0.08^a^	0.30^bc^	0.18^ab^	0.34^c^	0.03	0.002
*G: Lachnospiraceae XPB1014 group*	0.12^b^	0.18^a^	0.04^a^	0.17^a^	0.02	0.007
*G: Acetitomaculum*	0.27	0.38	0.39	0.33	0.03	0.39
*G: Syntrophococcus*	0.10	0.11	0.12	0.06	0.01	0.13
P: Firmicutes; F: Anaerovoracaceae						
*F: Anaerovoracaceae*	0.54	0.70	0.43	0.54	0.04	0.06
*G: Mogibacterium*	0.42^b^	0.31^a^	0.25^a^	0.23^a^	0.02	0.01
*G: Family XIII AD3011 group*	0.06^a^	0.20^b^	0.09^a^	0.09^a^	0.02	0.004
*G: Anaerovorax*	0.03^ab^	0.06^b^	0.04^b^	0.10^b^	0.01	0.002
F: Acholeplasmataceae*;*	0.03	0.05	0.03	0.04	0.01	0.43
*G: Anaeroplasma*						
F: Acidaminococcaceae;	0.03	0.03	0.02	0.03	0.00	0.12
*G: Succiniclasticum*						
F: Monoglobaceae;	0.02	0.12	0.06	0.05	0.02	0.19
*G: Monoglobus*						
P: Planctomycetota						
F: Pirellulaceae;	0.26^c^	0.07^b^	0.02^a^	0.04^ab^	0.02	0.0001
*G: p-1088-a5 gut group*						
P: Proteobacteria						
F: Enterobacteriaceae;	4.29^b^	0.03^a^	0.06^a^	0.04^a^	0.44	0.0001
*G: Escherichia-Shigella*						
P: Spirochaetota; F: Spirochaetaceae						
*G: Sphaerochaeta*	5.38^b^	0.81^a^	1.95^a^	0.43^a^	0.62	0.007
*G: Treponema*	0.01	0.04	0.04	0.06	0.01	0.44

P = phylum; F = family; G = genus; C = goats supplemented with control diet; A10= goats supplemented 10% azolla; A20 = goats supplemented with 20% azolla; A30 = goats supplemented with 30% azolla; ^a,b,c,d^ Means within a row with different subscripts differ significantly (p < 0.05). SEM = Standard error of means.

Phylum Firmicutes represented the second abundant phylum and exhibited statistically significant differences among treatments (p < 0.05), with the highest relative abundances observed in A10 and A30, followed by A20, and the lowest in the C group (Table 2). The phylum Firmicutes was classified into families Christensenellaceae, Ruminococcaceae, Oscillospiraceae, Hungateiclostridiaceae, Lachnospiraceae, Anaerovoracaceae, Acholeplasmataceae, Acidaminococcaceae, and Monoglobaceae (Table 3). Genus *Christensenellaceae R-7 group* within Christensenellaceae was higher Azolla treatments and found in order 2.97<2.95<2.60<1.88for groups A20, A10, A30, and C, respectively (Table 3) (p<0.05). The members of family Ruminococcaceae and Oscillospiraceae were higher in Azolla-supplemented groups (p<0.05). Family Lachnospiraceae was higher in Azolla-supplemented groups (p<0.05) and dominated by genus *Butyrivibrio* (Table 3). Genus *Saccharofermentans* dominated Family Hungateiclostridiaceae and had higher relative abundance in group C (0.35%) and A10 (0.32%) compared to A20 (0.14%) and A30 (0.16) (p < 0.05).

The phylum Planctomycetota was affiliated with the family Pirellulaceae and genus *p-1088-a5 gut group* that was decreased in supplemented groups (p<0.05) (Table 3). The phylum Proteobacteria was dominated by family Enterobacteriaceae and genus *Escherichia-Shigella,* which was higher in the control group (4.29%) compared to A10 (0.03%), A20 (0.06%), and A30 (0.04%) (P<0.05) (Table 3). Phylum Spirochaetota was dominated by family Spirochaetaceae and genus *Sphaerochaeta*, which was higher in the control group (5.38%), A10 (0.81%), A20 (1.95%), and A30 (0.43%) (Table 3) (p<0.05).

### Digestibility and rumen fermentation

Feed intake (g/kg0.75) of DM, OM, CP, CF, EE, NDF, and ADF were similar between experimental groups (p<0.05) (Table 4). Including the Azolla in goats’ diets did not affect the digestibility of nutrients except for crude protein digestibility (CPD), which showed its lowest value in goats fed A30 (63.93%) compared to C (74.76%), A10 (69.11%), and A20 (63.93%) (p<0.05) (Table 4). Digestible crude protein (DCP) followed the same trend. Moreover, higher numeric digestibility of crude fiber (CFD) was observed in group A20 without a significant difference (p>0.05) (Table 4). Azolla supplementation affected the rumen fermentation parameters. Total VFA was higher in supplemented groups (A10=106.04, A20=102.33, A30=105.32 mM) compared to the control (60.27 mM). Acetic, propionic, and butyric acids followed the same trend. (p<0.05) (Table 4). Higher isobutyric was observed in groups C and A30, while lower values were observed in groups A10 and A20 (p<0.05) (Table 4). Predicted methane (g /kg DMI) was lower in Azolla-supplemented groups compared with the control group (C= 39.87, A10= 25.38, A20= 25.14, A30= 24.34 g /kg DMI) (p<0.05) (Table 4).

**Table 4:** Effect of Azolla supplementation on the digestibility of nutrients and rumen fermentation parameters of goats.

	C	A10	A20	A30	SEM	p-value
	**Mean**	**Mean**	**Mean**	**Mean**		
Weight, kg	39.00	38.10	40.10	36.90	1.07	0.78
Feed intake, g/kg^0.75^						
Dry matter intake (DMI)	56.48	52.46	50.69	52.60	1.43	0.56
Organic matter intake (OMI)	47.80	45.49	43.56	43.13	1.04	0.38
Crude protein intake (CPI)	8.47	8.05	7.73	7.69	0.18	0.43
Crude fiber intake (CFI)	9.59	9.41	9.45	9.83	0.21	0.89
Ether extract intake (EEI)	2.10^b^	1.84^ab^	1.83^a^	1.94^ab^	0.04	0.015
Neutral detergent fiber intake (NDFI)	23.20	23.83	24.39	26.00	0.56	0.34
Acid detergent fiber intake (ADFI)	14.68	14.77	14.84	15.17	0.42	0.96
Digestion coefficients, %						
Dry matter digestibility (DMD)	67.82	66.28	66.55	63.46	0.70	0.15
Organic matter digestibility (OMD)	70.65	69.57	70.18	67.30	0.63	0.25
Crude protein digestibility (CPD)	74.76^b^	69.11^b^	68.24^b^	63.93^a^	1.05	0.0001
Crude fiber digestibility (CFD)	34.37	34.70	39.11	37.88	1.23	0.46
Ether extract digestibility (EED)	68.73	56.93	65.45	55.85	2.55	0.20
Nitrogen-free extract digestibility (NDFD)	55.47	59.02	61.06	57.86	1.04	0.29
Acid detergent fiber digestibility (ADFD)	53.62	56.01	58.05	49.30	1.30	0.09
Nutritive values, %						
Digestible crude protein (DCP)	12.11^b^	11.43^b^	11.08^b^	10.42^a^	0.15	0.009
Total digestible nutrients (TDN)	53.52	54.42	53.33	50.05	0.68	0.12
Rumen fermentation parameters						
PH	5.92	5.96	6.14	6.12	0.06	0.53
Ammonia (mg/100 mL)	6.00	5.86	5.62	5.53	0.12	0.53
Acetic, mM	40.78^a^	76.06^b^	72.38^b^	70.62^b^	3.79	0.0001
Propionic, mM	9.38^a^	15.51^b^	15.51^b^	15.89^b^	0.77	0.001
Iso butyric, mM	1.57^b^	0.75^a^	0.90^a^	1.40^bc^	0.12	0.024
Butyric, mM	5.69^a^	11.97^b^	11.52^b^	14.58^b^	0.96	0.002
Iso valeric, mM	1.96	0.80	1.13	1.90	0.19	0.08
Valeric, mM	0.90	0.93	0.89	0.91	0.04	0.98
Total VFA, mM	60.27^a^	106.04^b^	102.33^b^	105.32^b^	5.25	0.0001
Predicted methane, g /kg DMI	39.87^b^	25.38^b^	25.14^b^	24.34^a^	1.85	0.001

C= goats supplemented with control diet; A10= goats supplemented 10% azolla; A20= goats supplemented with 20% azolla; A30=goats supplemented with 30% azolla; VFA = Volatile fatty acids; mM = millimolar; ^a,b,c,d^ Means within a row with different subscripts differ significantly (p < 0.05). SEM = Standard error of means.

### Milk yield and feed efficiency

Milk yield and fat-corrected milk were significantly affected by Azolla supplementation (p < 0.05). Fat-corrected milk was 992.3, 1050.4, 1139.6, and 888.9 g/head/day in C, A10, A20, and A30 with change by +5.8%, +14.9%, and -10.5% in groups A10, A20, and A30 compared to control group (Table 5)

**Table 5:** Effect of Azolla supplementation on daily milk production, composition, and feed conversion ratio of goats.

	C	A10	A20	A30	SEM	p- value
	**Mean**	**Mean**	**Mean**	**Mean**		
Weight	42.00	40.42	42.00	40.00	0.93	0.21
DMI, g/h/d	1600	1550	1600	1550	21.96	0.99
Milk yield						
Milk yield g/h/d	1229.40^b^	1243.40^bc^	1341.70^c^	1103.80^a^	24.60	0.001
Fat corrected milk, g/h/d	992.25^ab^	1050.42^bc^	1139.61^c^	888.90^a^	26.80	0.002
Milk chemical composition, %						
Fat	2.70	2.98	3.03	2.70	0.07	0.25
Protein	2.97	2.97	2.69	2.86	0.07	0.46
Lactose	4.72	4.72	4.38	4.45	0.07	0.15
SNF	8.46	8.45	7.81	8.04	0.12	0.15
Ash	0.77	0.76	0.74	0.75	0.007	0.22
T.S	11.16	11.43	10.81	10.67	0.15	0.27
Feed conversion						
Milk yield, g/kg DMI	770^b^	802^bc^	838^c^	712^a^	0.012	0.00
Fat corrected milk g/kg DMI	620^b^	682^bc^	712^c^	576^a^	0.015	0.001
Fat g/kg DMI	20.77^ab^	23.88^bc^	25.13^c^	19.02^a^	0.75	0.004

^a,b,c,d^ Means within a row with different subscripts differ significantly (p < 0.05). C= goats supplemented with control diet; A10= goats supplemented 10% azolla; A20= goats supplemented with 20% azolla; A30=goats supplemented with 30% azolla.

SEM= Standard error of means; TS= Total solids; SNF= Solid non-fate; DMI = Dry matter intake.

Feed conversion efficiency based on milk yield and fat-corrected milk (g/kg DMI) was higher group A20 and lower in A30 compared to other groups and found in order A30<C<A10<A20 . Fat yield g/kg DMI followed the sam trend. Milk chemical composition (fat, protein, lactose, SNF, ash, and total solids) was not significantly affected by Azolla supplementation (p > 0.05). **PCA and Bray Curtis PERMANOVA analyses**

**Fig. 2:**
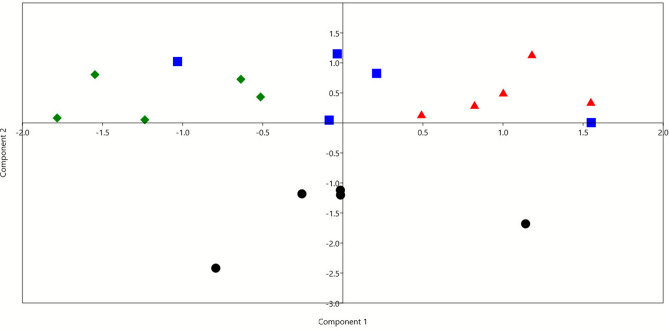
Principal component analysis (PCA) was determined using the results of milk yield, digestibility, rumen fermentation parameters, and relative abundances of rumen bacteria. The black dots are for goats supplemented with the control diet (C), the blue squares are for goats supplemented with 10% azolla (A10), the red triangles are for goats supplemented with 20% azolla (A20), and the green diamonds are for goats supplemented with 30% azolla (A30).

Multivariate analysis was conducted using PCA based on milk yield, nutrient digestibility, rumen fermentation parameters, and the relative abundances of rumen bacteria (Figure 2). The PCA revealed distinct clustering of samples according to the nutritional treatments, primarily driven by variations in milk yield, methane production, total VFA, and the relative abundances of Bacteroidota, Firmicutes, and the *Rikenellaceae RC9 gut group*. PERMANOVA confirmed that these differences among groups were statistically significant (p = 0.0005). Pairwise comparisons with Bonferroni correction showed significant differences between the control (C) and A20 (p = 0.02), C and A30 (p = 0.01), A10 and A30 (p = 0.02), and A20 and A30 (p = 0.008), indicating that increasing levels of Azolla supplementation significantly influenced both rumen microbial composition and associated fermentation and production parameters.

### Pearson correlation analysis

Pearson correlation analysis (Supplementary Figure S2) revealed both positive and negative associations among measured parameters. Milk yield was positively correlated with dry matter digestibility (DMD) and the relative abundance of the phylum Bacteroidota. Additionally, DMD showed a positive correlation with the abundance of the Rikenellaceae RC9 gut group, highlighting potential links between rumen microbial composition, nutrient utilization, and milk production.

## Discussion

### Nutritive value of Azolla

Azolla is a promising unconventional feed resource due to its rich nutritional profile (Table 1) and abundance of bioactive compounds. In the present study, total phenols and tannins in Azolla (Supplementary Table S1) were comparable to the range reported by Tran et al.^30^. Whereas Kösesakal and Yıldız^31^ observed higher concentrations of total phenols, tannins, and flavonoids than those obtained in the current results. Nevertheless, Azolla contains significantly higher levels of key phytochemicals compared to conventional feed ingredients such as Berseem hay and CFM. Phytochemical profile is likely a major factor influencing ruminal fermentation dynamics and microbial diversity, ultimately contributing to improved animal productivity, and its incorporation into diets at different levels^10^, since diet composition is the primary driver of the rumen microbial community and fermentation^13^. To the best of our knowledge, this is the investigation of effect of Azolla supplementation on the modulation of rumen bacteria and its relation to the performance of lactating goats.

### Effect of Azolla on the modulation of microbial community

Higher microbial diversity was associated with Azolla-supplemented goats (Table 2). Similar findings were obtained in sheep fed *Ulva spp* or microalgae^32,33^. Higher microbial diversity could be attributed to the diverse nutrients and bioactive compounds in the azolla^10,33^. Higher microbial diversity boosts the energy metabolism and forage digestion^34^). In this trial, Bacteroidota and Firmicutes emerged as the dominant phyla, which is consistent with findings of an *in vitro* study on diets containing different levels of Azolla ^10^, goats supplemented with phytochemicals^13^, and lambs received *Ulva* spp^33^.

Phylum Bacteroidota was dominated by the genera *Prevotella* and *Rikenellaceae RC9* gut group (Table 3). Similar findings were obtained by an *in vitro* study on azolla and goats fed different phytochemical sources^10,13^. Genus *Prevotella* showed a consistent increase with Azolla supplementation (Table 3). This enrichment agrees with phytochemical-rich and microalgae-based diets^35^. Functionally, *Prevotella* is recognized for its versatile metabolic capacity, including the degradation of soluble carbohydrates and proteins^36,37^. Its expansion in the current study suggests that Azolla supplementation may stimulate different fermentation pathways, thereby enhancing overall ruminal fermentation efficiency. Furthermore, *Prevotella* tolerate some types of phytochemicals such as tannins^13^, which demonstrate their prevalence in supplemented groups. The *Rikenellaceae RC9 gut group* revealed lower proportions in Azolla-supplemented groups (Table 3). This genus was higher in cows supplemented with a lower dose of *Phyllanthus emblica*, which is rich in phytochemicals^38^, indicating that this genus affords a lower content of phytochemicals. Genus *Rikenellaceae RC9 gut group* converts fiber to acetic and propionic acids, succinate, which consume hydrogen from rumen and decreases methane production^39^.

Firmicutes exhibited a consistent and significant rise in Azolla-fed groups (Table 2). Similarly, Shinkai et al.^40^ reported that tannin supplementation increased the Firmicutes/Bacteroidetes ratio, mainly through the selective promotion of *Ruminococcaceae* over members of the genus *Prevotella*. These findings agree with previous studies that reported the effect of dietary phytochemicals on rumen bacteria^10,13,41^. Phylum Firmicutes was enriched with the members of family Ruminococcaceae and genus *Christensenellaceae R-7 group,* which were higher in the Azolla-supplemented groups (Table 3), indicating that they resist phytochemicals in Azolla^13^. Family *Ruminococcaceae* and genus *Christensenellaceae R-7 group* have a pivotal role in fiber degradation and produce different VFAs such as acetic and butyric, which improve feed conversion efficiency in ruminants^42,43^. Collectively, these findings suggest that Azolla supplementation fosters a more fibrolytic and functionally stable rumen microbiome, thereby enhancing fiber utilization efficiency.

In contrast, opportunistic or dysbiosis-associated genera such as *Escherichia-Shigella* and *Sphaerochaeta* were significantly reduced in Azolla-supplemented goats (Table 3). These taxa have been associated with reduced rumen stability and suboptimal fiber metabolism, as *Escherichia-Shigella* is a potential pathogen known to delay the establishment of the anaerobic rumen environment^44^. Notably, our results are consistent with those of Ahmed et al.^45^, who similarly reported negative associations of *Escherichia-Shigella* with fibrolytic taxa and positive correlations among beneficial fiber-degrading genera.

The decline in some microbial genera due to Azolla supplementation could be attributed to the negative effect of phytogenic compounds such as tannins and saponins that disrupt the microbial cell wall^10,13,46^.

### Effect of Azolla on feed utilization and rumen fermentation

Changes in the rumen bacteria driven by Azolla supplementation are closely associated with modifications in fermentation characteristics and nutrient digestibility (Table 4). The decrease in the CPD in goats fed a higher level of Azolla (A30) could be attributed to the elevated content of plant secondary metabolites, particularly tannins and phenolic compounds, which possess antimicrobial properties and can form complexes with dietary proteins and carbohydrates, thereby limiting their availability to rumen microbes and reducing the protein digestion^10,13,46^. Moreover, the increased dietary ash and fiber with higher levels of dietary Azolla, may further depress digestibility (Table 1 and 3), likely due to inert ash fractions that dilute dietary energy and interfere with microbial fermentation^47^.

Higher fiber digestibility in A20 , was associated with the enrichment of fiber-degrading microbes, including *Prevotella*, Ruminococcaceae, and *Christensenellaceae R-7 group,* which agrees with previous studies^13,48–51^. Additionally, micro-elements and vitamins support rumen microbiota and are required for efficient digestion, absorption, and metabolism^49,50^.

These microbial shifts explain the improved fiber digestibility, higher VFA production, and better nutrient utilization observed with Azolla supplementation, especially groups A10 and A20. Similar improvements were reported in lactating goats fed 10% Azolla^11^. Similarly, Nayel et al.^52^ demonstrated that Azolla extract enhanced nutrient digestibility and nitrogen balance in Ossimi sheep. Collectively, these findings confirm that phytogenic feed additives can beneficially modulate rumen microbial fermentation, improve host metabolism, and enhance ruminant performance, while contributing to CH₄ mitigation through suppressing main rumen methanogens^13,53,54^. These findings agree with several *in vivo* studies, which recommended 10–20% Azolla inclusion as optimal for improving nutrient digestibility, fermentation characteristics, and overall nutritional value in ruminants.^11,52,55^.

Additionally, these findings demonstrate that these Azolla supplementation levels (with it’s phytochemicals content) favor bacterial groups that have central roles in the rumen fermentation, such as family *Ruminococcaceae* and *Prevotellaceae,* which resulted in higher VFA synthesis^56–58^. In the same line, lactating goats supplemented with 10% Azolla showed improved nutrient digestibility and higher VFA production^11,52^. In line with these findings, enhanced VFA production was also reported in growing goats supplemented with a phytogenic mixture at 1% of DM intake^13^ and in buffalo supplemented with flavonoids^59^, highlighting that moderate levels of phytogenic compounds can ensure beneficial impacts on rumen fermentation and overall animal performance.

The proportion of individual VFAs shifted, with acetate remaining the predominant fraction, followed by propionate and butyrate. Notably, the increased acetate-to-propionate ratio at moderate levels of Azolla inclusion (10–20%) reflected enhanced fiber fermentation, consistent with the higher relative abundance of fiber-degrading bacteria. Lower methane in Azolla-supplemented groups agrees with findings on buffalo supplemented with an herbal mixture^50^. Lower methane improves animal efficiency as methane represents a loss of 2-12 % of gross energy feed intake^50,51^. The lower methane is associated with a higher proportion of propionic-producing bacteria, such as *Prevotella*, and rumen propionic acid production. The propionic generation absorbs the rumen hydrogen, which suppresses the methanogenesis^13,50,51^. Additionally, phytochemicals such as tannins and saponins suppress rumen methanogens through disrupting the cell wall and inhibiting the growth and propagation of rumen methanogens^13^.

### Impact of Azolla on milk production and feed efficiency

Milk yield was increased markedly in the A10 and A20 groups (Table 5), while a significant decline was recorded in A30. These outcomes align with the observed improvements in rumen VFAs, which are principal energy precursors for ruminants^13,51^. Similar findings were obtained in lactating goats and ewes^54,60,61^. Recent studies support these findings, where goats fed diets containing 5–10% Azolla showed significant improvements in milk yield and fat content^11,62^. Phytochemicals in Azolla enhance animal health by boosting immunity and antioxidant capacity, which improves feed conversion^63^. Furthermore, propionic acid is a precursor for gluconeogenesis and lactose synthesis, which increase milk yield^64^.

In conclusion, the dietary inclusion of Azolla as a partial replacement for concentrate feed mixture of lactating goats, enhanced fiber-degrading bacteria in the rumen, which improved fiber utilization and VFA production. Consequently, milk yield was improved. Higher Azolla inclusion level, 30%, depressed the digestibility of protein and milk yield. Subsequently, inclusion of Azolla at 20% on concentrates mixture of lactating goats’ diet, guarantees the positive effect on the rumen ecosystem and animal performance and represent a feasible alternative of conventional feed ingredients. This study was constrained by limited animal numbers to be used to increase the azolla inclusion levels on for longer period and in different lactation stages.

## Supplementary Information

Supplementary Information 1.

Supplementary Information 2.

Supplementary Information 3.

## Data Availability

The raw sequence reads are available at: https:/www.ncbi.nlm.nih.gov/sra/PRJNA1291228.
