# Supplementary material for: Rumen bacteria, feed utilization, and milk production of Damascus goats fed different levels of azolla meal
Source: Sci Rep. 2026 Apr 23;16:13279. doi: 10.1038/s41598-026-38113-6 (PMC13106777; doi:10.1038/s41598-026-38113-6)
Supplement: Supplementary file 1 — Supplementary Information 1. [file 41598_2026_38113_MOESM1_ESM.pdf]

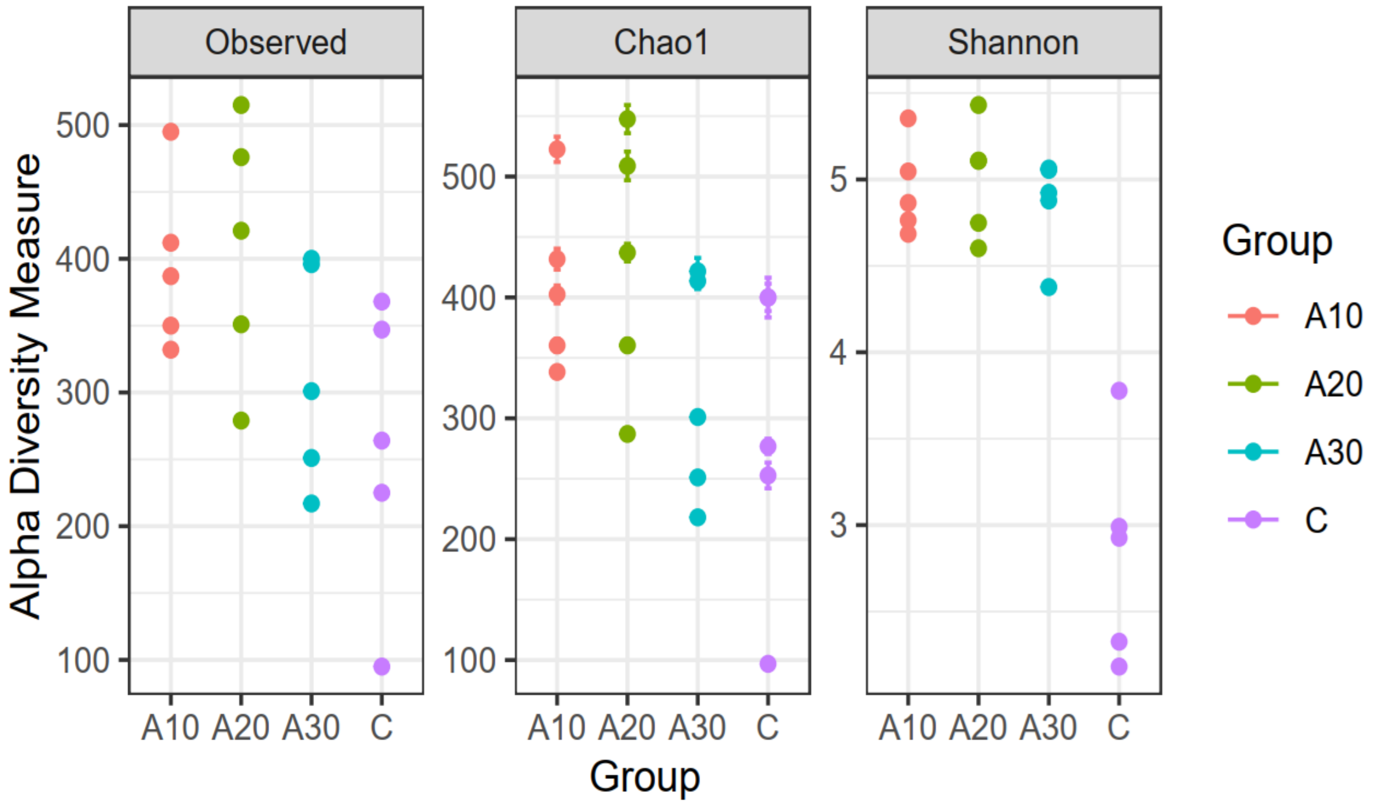

Supplementary Figure S1: Alpha diversity of microbial communities in the rumen of lactating goats supplemented with different levels of Azolla. Purple circles for goats supplemented with control diet (C), red circles for goats supplemented with 10% azolla (A10), green circles for goats supplemented with 20% azolla (A20), and blue circles for goats supplemented with 30% azolla (A30).
