# Supplementary material for: Rumen bacteria, feed utilization, and milk production of Damascus goats fed different levels of azolla meal
Source: Sci Rep. 2026 Apr 23;16:13279. doi: 10.1038/s41598-026-38113-6 (PMC13106777; doi:10.1038/s41598-026-38113-6)
Supplement: Supplementary file 2 — Supplementary Information 2. [file 41598_2026_38113_MOESM2_ESM.docx]

Supplementary Table S1: The phytochemical content in the animal feeds.

|  | **CFM** | **Berseem hay** | **Azolla** |
| --- | --- | --- | --- |
| **Polyphenols %** | 0.05 | 0.09 | 5.00 |
| **Flavonoids%** | 0.40 | 0.31 | 0.23 |
| **Tannins %** | 0.17 | 0.18 | 4.31 |
|  | **Phenols and flavonoids, µg/g** | | |
| **Gallic acid** | 1.50 | 00 | 300.14 |
| **Chlorogenic acid** | 0.10 | 00 | 1246.64 |
| **Methyl gallate** | 00 | 00 | 8.94 |
| **Caffeic acid** | 00 | 00 | 242.29 |
| **Ellagic acid** | 00 | 0.80 | 134.72 |
| **Vanillin** | 00 | 00 | 261.26 |
| **Rosmarinic acid** | 00 | 00 | 588.94 |
| **Resorcinol** | 0.80 | 00 | 1.00 |
| **Catechin** | 00 | 00 | 106.19 |
| **Rutin** | 00 | 15.00 | 162.22 |
| **Naringenin** | 00 | 00 | 1574.56 |
| **Daidzein** | 00 | 00 | 2.58 |
| **Quercetin** | 00 | 7.00 | 6.97 |
| **Kaempferol** | 00 | 50.00 | 1.31 |
| **Hesperetin** | 00 | 00 | 7.70 |
| **Hesperidin** | 00 | 00 | 295.00 |
| **Apigenin** | 00 | 10.00 | 21.7 |
| **Phenanthrene** | 0.40 | 3.00 | 12.00 |
| **Pyrocatechol** | 0.40 | 00 | 23.80 |
| **Coumaric acid** | 00 | 50 | 23.08 |
| **Ferulic acid** | 0.20 | 0.10 | 1.00 |
| **Cinnamic acid** | 2.90 | 0.50 | 1.51 |
| **Diosmin** | 00 | 30.00 | 00 |
| **Quinic** | 13.70 | 5.00 | 00 |
