# Supplementary material for: Rumen bacteria, feed utilization, and milk production of Damascus goats fed different levels of azolla meal
Source: Sci Rep. 2026 Apr 23;16:13279. doi: 10.1038/s41598-026-38113-6 (PMC13106777; doi:10.1038/s41598-026-38113-6)
Supplement: Supplementary file 3 — Supplementary Information 3. [file 41598_2026_38113_MOESM3_ESM.pdf]

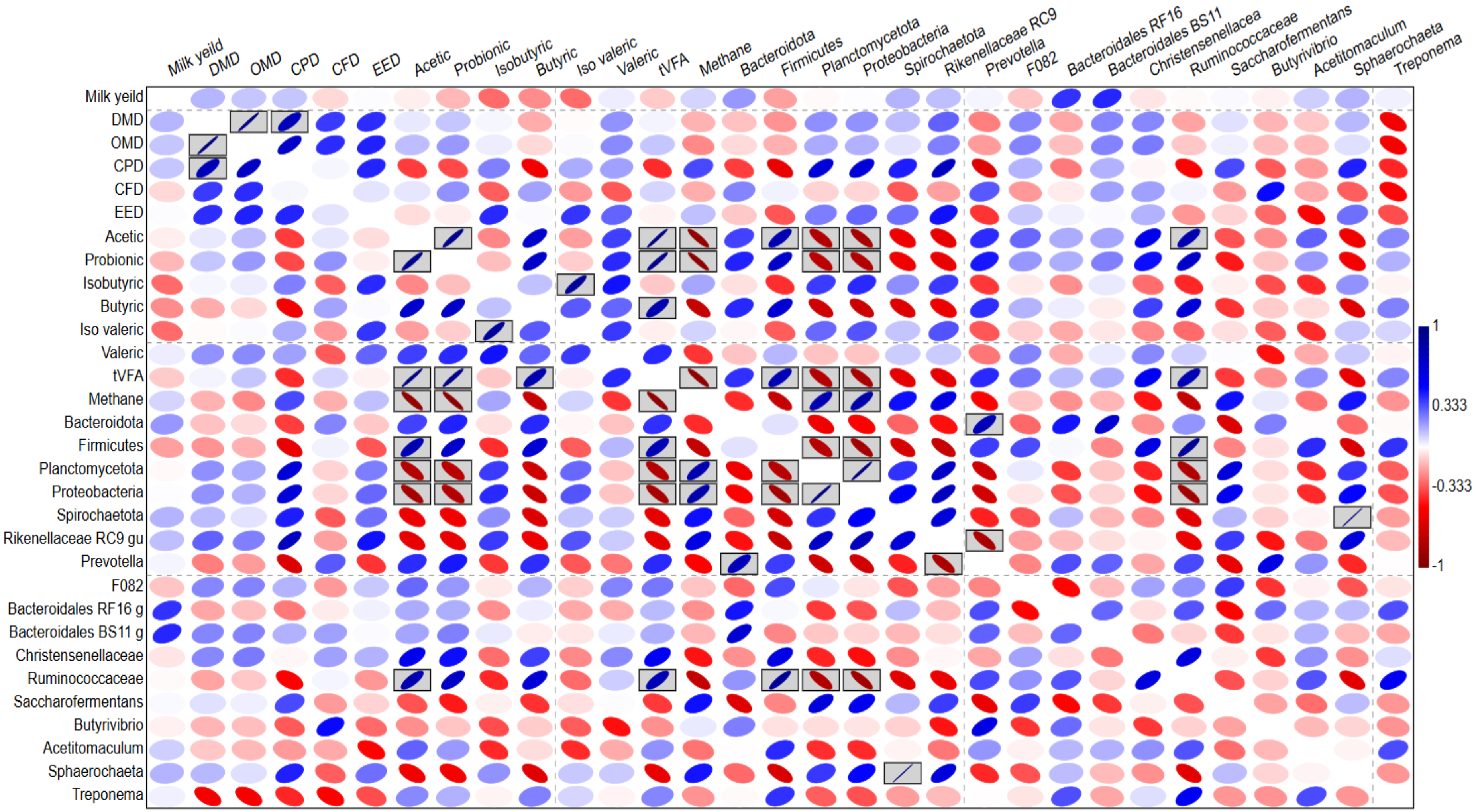

Supplementary Figure S2: Heatmap based on Pearson correlation. The correlation was conducted using the data milk yield, the relative abundance of dominant bacteria, digestibility of nutrients, and rumen fermentation parameters of goats supplemented with different levels Azolla. The black boxed ellipses indicate to significant correlations at $P < 0.05$
